# Unilateral Exoskeleton Imposes Significantly Different Hemispherical Effect in Parietooccipital Region, but Not in Other Regions

**DOI:** 10.1038/s41598-018-31828-1

**Published:** 2018-09-07

**Authors:** Junhua Li, Nitish Thakor, Anastasios Bezerianos

**Affiliations:** 1 Singapore Institute for Neurotechnology (SINAPSE), Centre for Life Sciences, National University of Singapore, Singapore, 117456 Singapore; 2 Laboratory for Brain-bionic Intelligence and Computational Neuroscience, Wuyi University, Jiangmen, 529020 China; 3 Centre for Multidisciplinary Convergence Computing (CMCC), School of Computer Science and Engineering, Northwestern Polytechnical University, Xi’an, 710072 China

## Abstract

In modern society, increasing people suffering from locomotor disabilities need an assistive exoskeleton to help them improve or restore ambulation. When walking is assisted by an exoskeleton, brain activities are altered as the closed-loop between brain and lower limbs is affected by the exoskeleton. Intuitively, a unilateral exoskeleton imposes differential effect on brain hemispheres (i.e., hemispherical effect) according to contralateral control mechanism. However, it is unclear whether hemispherical effect appears in whole hemisphere or particular region. To this end, we explored hemispherical effect on different brain regions using EEG data collected from 30 healthy participants during overground walking. The results showed that hemispherical effect was significantly different between regions when a unilateral exoskeleton was employed for walking assistance and no significance was observed for walking without the exoskeleton. Post-hoc t-test analysis revealed that hemispherical effect in the parietooccipital region significantly differed from other regions. In the parietooccipital region, a greater hemispherical effect was observed in beta band for exoskeleton-assisted walking compared to walking without exoskeleton, which was also found in the source analysis. These findings deepen the understanding of hemispherical effect of unilateral exoskeleton on brain and could aid the development of more efficient and suitable exoskeleton for walking assistance.

## Introduction

A wide range of causes, such as stroke, traffic accident, and aging, can result in locomotor deficit or disability^1–3^. The lack or loss of locomotion significantly restricts one’s participation in daily living, and might require caregivers to assist in some cases. In order to restore the movement function of these disable people and improve their independence, exoskeletons are prevalently utilized to either restore one’s own motor function by rehabilitation training or regain mobility with exoskeleton assistance^4^. There is evidence to support that exoskeletons are applicable and useful in the aforementioned applications^5–7^.

In order to investigate the effect of exoskeleton on human body and mind, multi-modal signals are recorded while people are moving, namely mobile brain/body imaging (MoBI)^8^. When an exoskeleton is used, it leads to changes of gait pattern and alterations in brain activity. It has been shown that kinematic and kinetic characteristics are changed due to the use of exoskeleton. Adamczyk and Kuo investigated gait features (e.g., step time and step length) for a cohort of exoskeleton-assisted amputees, and found that gait asymmetry was present in their walking^9^. The influence of exoskeletons was also observed in a trajectory comparison study, showing that hip and ankle extension and motion range in the case of using gait-orthosis were significantly greater than that of walking without gait-orthosis^10^. Besides, the assistive torque provided by an exoskeleton influences the energy expenditure in muscular contraction, resulting in a change of activation patterns of muscles^11^. This effect could also be upstream passed to the central nervous system and gives rise to alterations of brain activities, exhibiting a certain correlation between electromyogram (EMG) and electroencephalogram (EEG)^12^. Another study addressed that the EEG-EMG correlation was increased when a stronger muscular contraction was performed^13^. The mainly involved frequency bands are alpha and beta, which are repeatedly reported in literature^14–18^. For instance, Severens and his colleagues found that brain wave oscillations in alpha and beta bands were desynchronized during walking^19^. This desynchronization information can be extracted as features to classify different walking conditions. The feasibility of classification of walking conditions based on EEG features was corroborated in a recent study^20^. In addition, brain activity is time-locked to the gait cycle, showing that walking-related activation is pronounced in slightly different areas for different phases of gait cycle^21^. These areas are mainly located in the sensorimotor cortex, which is considered as a locomotion-related region^22^. Nonetheless, other regions, such as the frontal cortex, were also found to be relevant to walking^18^.

As we know, limbs are significantly linked to contralateral hemispheres, displaying that limb movement at one side activates the other hemisphere of the brain. The corresponding cortex of lower limbs is closer to the brain midline compared to the corresponding cortex of upper limbs, but the contralateral control mechanism is identical. This mechanism of contralateral control could lead to differential effect on hemispheres (i.e., hemispherical effect) when the engagement of two lower limbs is different due to the use of unilateral exoskeleton. It induces us to consider whether the extent of hemispherical effect is identical across brain regions. If not, which region does the hemispherical effect appear in? To address the questions, we analysed the data collected from a cohort of healthy participants who performed walking with and without a unilateral exoskeleton, and compared hemispherical effect between these two walking conditions. Due to the fact that walking is mostly related to alpha and beta bands^14–17^, this study focused on these two bands for exploring hemispherical effect. To the best of our knowledge, this study is the first to investigate hemispherical effect of spectral power density with respect to exoskeletal walking assistance. The hemispherical effects on regions and frequency bands are revealed by both region-based and electrode-based explorations. The results derived from this study are meaningful to the applications of the unilateral exoskeleton because its effect on different hemispheres can be taken into consideration to guide design, manufacture, and usage of the next generation of exoskeleton.

## Results

**Figure 1:**
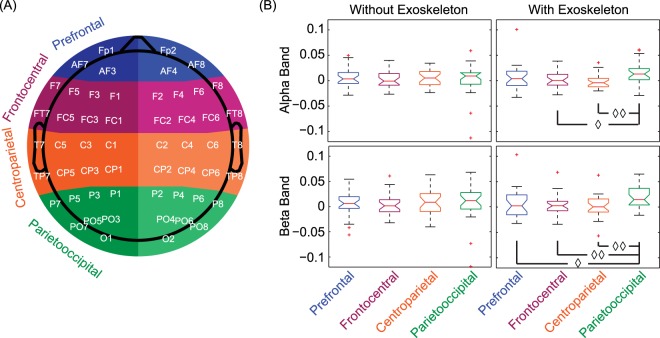
(**A**) The layout of electrodes used for EEG measurement and the illustration of area partition. (**B**) Statistical results of one-way analysis of variance (ANOVA) and post-hoc two-tailed paired t-test. The ANOVA results show that there is no significant difference in power asymmetry between cortical regions for both alpha and beta bands when participants walked without exoskeleton, while the significant difference was observed in power asymmetry for both bands when an exoskeleton was used to assist the walking. The post-hoc two-tailed paired t-test indicates that power asymmetry in the pariatooccipital region is significantly larger than that of other regions (◊ stands for p < 0.05, and ◊◊ stands for p < 0.005).

Significant hemispherical effect was observed under the condition of using a unilateral exoskeleton in both region-based and electrode-based cases. In the region-based case, one-way analysis of variance (ANOVA) showed that there was a statistically significant difference between the means of power asymmetry indices of different regions (i.e., prefrontal, frontocentral, centroparietal, and parietooccipital regions) for both alpha band [F(3,104) = 3.32, p = 0.023] and beta band [F(3,104) = 3.64, p = 0.015], when a unilateral exoskeleton was utilized to assist walking. This significant difference was not present for the condition of walking without the exoskeleton [F(3,104) = 0.19, p > 0.05 for alpha band, and F(3,104) = 0.14, p > 0.05 for beta band]. The post-hoc two-tailed paired t-test demonstrated that the power asymmetry index in parietooccipital region significantly differed from those in the other three regions (see Fig. 1(B)). In the electrode-based case, a higher number of electrode pairs with significant power asymmetry was found when an exoskeleton was used for walking assistance in both alpha and beta bands. However, only five electrode pairs in the beta band remained after multiple comparison correction (see Table 1). All remaining electrode pairs resided in parietooccipital region and exhibited leftward asymmetry.

**Table 1:** Statistics of power asymmetry for each pair of channels.

Pairs	Alpha Band				Beta Band			
	Without Exoskeleton		With Exoskeleton		Without Exoskeleton		With Exoskeleton	
	Original p-value	FDR-Corrected p-value	Original p-value	FDR-Corrected p-value	Original p-value	FDR-Corrected p-value	Original p-value	FDR-Corrected p-value
Fp1-Fp2	—	—	—	—	—	—	—	—
AF7-AF8	P = 0.034 ←	—	—	—	—	—	—	—
AF3-AF4	—	—	—	—	—	—	—	—
F7-F8	—	—	—	—	—	—	—	—
F5-F6	—	—	—	—	—	—	—	—
F3-F4	—	—	—	—	P = 0.035 ←	—	—	—
F1-F2	—	—	—	—	—	—	P = 0.032 ←	—
FT7-FT8	—	—	—	—	—	—	—	—
FC5-FC6	—	—	—	—	—	—	—	—
FC3-FC4	—	—	—	—	—	—	—	—
FC1-FC2	—	—	—	—	—	—	—	—
T7-T8	—	—	—	—	—	—	—	—
C5-C6	—	—	—	—	—	—	—	—
C3-C4	—	—	—	—	P = 0.023 ←	—	—	—
C1-C2	—	—	—	—	—	—	—	—
TP7-TP8	—	—	—	—	—	—	—	—
CP5-CP6	—	—	—	—	—	—	—	—
CP3-CP4	—	—	—	—	P = 0.029 ←	—	—	—
CP1-CP2	—	—	P = 0.014 →	—	—	—	—	—
P7-P8	—	—	P = 0.022 ←	—	—	—	—	—
P5-P6	—	—	P = 0.039 ←	—	P = 0.035 ←	—	P = 0.005 ←	P = 0.033 ←
P3-P4	—	—	—	—	—	—	P = 0.015 ←	—
P1-P2	—	—	—	—	—	—	—	—
PO7-PO8	P = 0.015 ←	—	P = 0.003 ←	—	P = 0.003 ←	—	P < 0.001 ←	P = 0.006 ←
PO5-PO6	P = 0.019 ←	—	P = 0.014 ←	—	P = 0.009 ←	—	P = 0.001 ←	P = 0.010 ←
PO3-PO4	—	—	P = 0.012 ←	—	—	—	P = 0.001 ←	P = 0.010 ←
O1-O2	—	—	P = 0.008 ←	—	—	—	P = 0.009 ←	P = 0.049 ←

^1^Two-tailed one-sample t-test.

^2^Represents no significance at the significance level of 0.05, ← indicates leftward asymmetry, → indicates rightward asymmetry.

**Figure 2:**
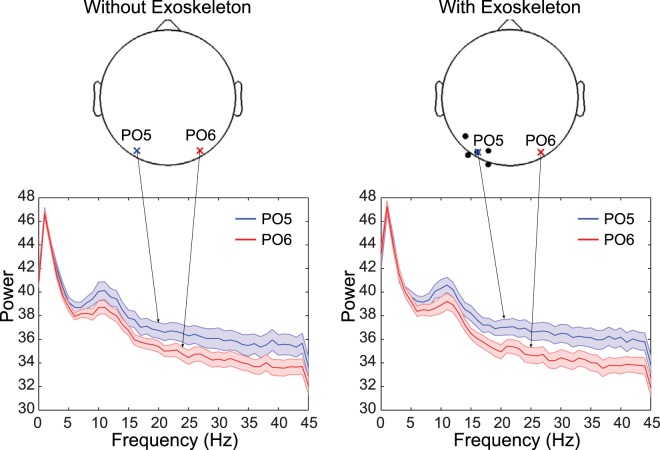
Channels of significant power asymmetry between hemispheres in the beta band and representative channels illustrating power spectral density (PSD). Black dots represent channels that appear significant power asymmetry between hemispheres (Only marked in the left hemisphere). The subplots at the bottom show the PSDs of the representative channels marked by crosses. The solid lines stand for the means averaged across subjects, and shaded areas indicate corresponding standard errors.

**Figure 3:**
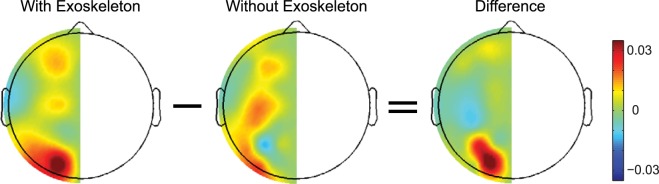
The difference of power asymmetry in the beta band between the conditions of with and without an exoskeleton. Power asymmetry was obtained by that the power in the right hemisphere was subtracted from the power in the left hemisphere and then normalized to the sum of powers of the left and right hemispheres. After that, the difference of power asymmetry between conditions was calculated by subtracting the power asymmetry of the condition without an exoskeleton from that of the condition with an exoskeleton.

We further explored power spectral density (PSD) at each frequency. Figure 2 illustrated an example of a representative channel pair. The PSD averaged across subjects in the left hemisphere is generally greater than that of the right hemisphere for both conditions (i.e., with and without an exoskeleton). However, the PSD difference between hemispheres is larger for the condition with the exoskeleton. This can explain why hemispherical power asymmetry is significant in beta band for the condition with exoskeleton, while there is no significance for the condition without the exoskeleton. Figure 3 shows the scalp distribution of power asymmetry difference between conditions in beta band. An obvious difference in localization is observed on the parietooccipital region. All other regions have small differences that do not reach a significance level of 0.05.

**Figure 4:**
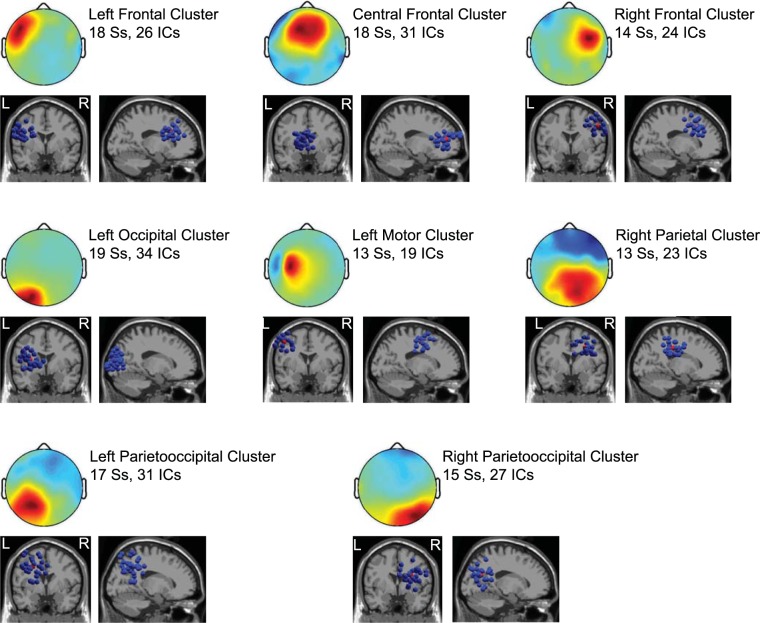
Walking-related Clusters. Cluster-mean scalp maps are shown at the upper rows while equivalent dipole source locations are shown at the bottom rows in each subplot. The numbers of independent components (ICs) and the numbers of subjects (Ss) to whom these ICs belong are described beside each scalp map.

**Figure 5:**
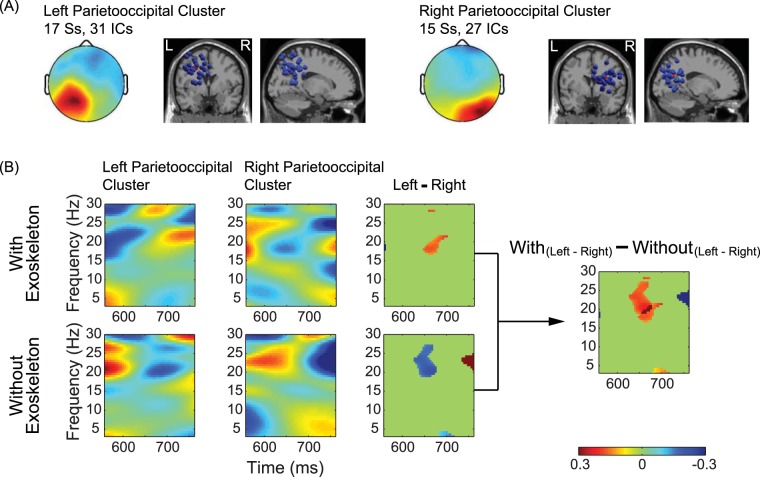
(**A**) Cluster-mean scalp map and equivalent dipole source locations. The left parietooccipital cluster consists of 31 independent components (ICs) from 17 subjects (Ss) and the right parietooccipital cluster consists of 27 ICs from 15 subjects. (**B**) Cluster-mean event-related spectral perturbation (ERSP) images for the conditions with and without an exoskeleton. The first and second columns in (**B**) shows grand normalized ERSP images for the left and right parietooccipital clusters, respectively. The spectral power averaged across all time points was subtracted from the corresponding spectral powers at each frequency. This was individually done for each gait cycle. These normalized ERSPs were then averaged across gait cycles to generate the grand normalized ERSP image. The third column shows the differences of ERSP between the left parietooccipital cluster and the right parietooccipital cluster. For the images in the third column, only significant parts are shown (p < 0.05, uncorrected). The rightmost image in (**B**) was obtained by that the upper image in the third column minuses the bottom image in the third column, illustrating the condition difference of the differences between left and right parietooccipital clusters.

The source clustering analysis revealed a few clusters related to walking (see Fig. 4). Two of them were located in the parietooccipital region (see Fig. 5(A)), where the power asymmetry difference was observed. The left parietooccipital cluster consists of 31 independent components (ICs) from 17 subjects (Ss) and the right parietooccipital cluster consists of 27 ICs from 15 Ss. The event-related spectral perturbation (ERSP) images of these two clusters show that the time-frequency representation is different between the left and right clusters. This difference is dependent on walking conditions, which is mostly in the beta band (see Fig. 5(B)).

## Discussions

Our study revealed that the use of an assistive unilateral exoskeleton influences brain activity and gives rise to significant hemispherical effect in the beta band in the parietooccipital region.

A previous study has found that exoskeleton-aided walking exhibited gait asymmetry in kinematic and kinetic measures, such as forward velocity^9^. The asymmetry in these kinematic and kinetic measures could also be related to foot orientation and waking posture^23^. All changes in these measures actually reflect a change in muscular activity. Therefore, some studies directly utilized EMG signal to investigate muscular activations during walking or jogging. Differential muscular activation between lower limbs was reported in a physical function study^24^. As we know, lower limb movement is supervised by the brain, which results in a close relationship between them. Accordingly, the existence of kinematic and kinetic asymmetry of lower limb movement could extrapolate the assumption that there is an asymmetric pattern in the brain. Our study gave evidence to support this assumption. We found that hemispherical effect exists in the parietooccipital region. The channel-based exploration also corroborated that only channel pairs in the parietooccipital region have significant hemispherical effect. It is worth noting that this region does not completely overlap the sensorimotor cortex that is a well-known region related to the movements of limbs. This might be because (1) despite the asymmetry in kinematic and kinetic measures and muscular activations of lower limb, these changes are not enough to broadly affect the corresponding brain cortex compared to the effect of movement itself; (2) the sensorimotor cortex might mainly involve in the output of movement commands, and might be relatively less for the input; (3) the afferent effect on the brain mostly influences visuospatial attention and visuomotor transformation. More spatial attention might be required to coordinate walking when participants wear an assistive exoskeleton, especially for those people who do not have any experience wearing exoskeletons. This requirement could lead to lateralization alteration on the occipital cortex, which is relevant to neural processing of movement planning^25^. In addition to the occipital cortex, the parietal cortex was also found to be involved in sensorimotor transformation from visual input to motor execution^26,27^. Both cortices might play an important role in the adaption of visual perception and processing of current gait posture with regard to an exoskeleton. These cortices are in agreement with the significantly asymmetric region observed in our present study. It might imply that the use of an exoskeleton gives rise to bigger influence on the neural processing of visuospatial attention and visuomotor transformation than on the neural processing of movement. As the findings derived from the comparison studies of voluntary movement and concurrent voluntary movement/functional electrical stimulation^28,29^, somatosensory cortex is an integration hub for processing information from both primary motor cortex output and the afferent input of proprioceptive feedback. This could explain why significant hemispherical difference (i.e., leftward asymmetry) was observed in the parietal region (somatosensory cortex resides in the parietal lobe) in our study. The afferent input of proprioceptive feedback produced the greater effect on the left hemisphere, which matches the experimental setting that a unilateral exoskeleton was mounted on the right lower limb only. This might be because more amount of proprioceptive information leads to stronger activation in the somatosensory cortex^30,31^.

In the exploration of source localization, a few walking-related clusters were found in the frontal, motor, parietal, and occipital cortices. This finding of widespread clusters is in agreement with the observation from a previous walking study^18^. Two of the clusters were located in the parietooccipital region where the power asymmetry difference was observed. The ERSP was different between these two clusters. This difference is dependent on walking conditions, which is mostly in the beta band. In this band, significant asymmetry was observed for the channel pairs located in the parietooccipital region (see Table 1). As shown in the region based results, hemispherical asymmetry indices of cortical regions were significantly different in both alpha and beta bands for the condition with exoskeleton. This finding is in line with the previous conclusion that the spectral powers of alpha and beta bands were modulated in the walking^15,16^. Besides the alpha and beta bands, the gamma band was also reported to be relevant to walking^32,33^. However, the opposite opinion stated that this might be due to artifact contamination^34^. Therefore, artifact removal is a crucial step for processing mobile EEG data. As shown in the study of Snyder *et al*., independent component based source localization can be a suitable method for mobile EEG data analysis due to its capability of isolating artifacts^35^. In this study, we utilized this method and found a few clusters that are in agreement with previous findings^18^. We also found that the between-walking-condition ERSP difference of the difference between the left parietooccipital cluster and the right parietooccipital cluster mostly appeared in the beta band. However, it is worth noting that these two parietooccipital clusters are not completely geometrically symmetric.

In our study, only male participants were recruited in the experiments due to the limit of the customized exoskeleton and non-equivalent strength of females (i.e., this confines the findings to the male population only). However, we speculate there is no significant difference between male population and female population, but further study is required for confirmation. In the further study, an adaptive exoskeleton is necessary in order to fit different strengths and heights. Alternatively, another exoskeleton can be customized for female population and employed to repeat the experiments. Results derived from the female population can then be compared to that of the male population.

The investigation of hemispherical effect is important not only for the understanding of neural mechanisms with respect to exoskeleton-aided walking, but also for the development of next generation exoskeletons that are more suitable for users. There is evidence indicating that asymmetric walking consumes more energy and consequently results in metabolic dissipation^36^. Similarly, hemispherical asymmetry in the brain could require more mental resources and expends more energy. When a new generation of exoskeleton is developed, hemispherical asymmetry index can be used to assess the effect derived from the exoskeleton on the brain. The lower hemispherical asymmetry index is, the less the effect of the exoskeleton imposes on the brain. Ideally, the hemispherical asymmetry should disappear even when an exoskeleton is used for walking assistance, similar to the case of walking without an exoskeleton. Such exoskeleton without the hemispherical asymmetry could benefit users by minimizing mental resource cost. The hemispherical asymmetry index is an added measure to the indicators based on kinematics and electromyogram for the assessment of a unilateral exoskeleton^37^.

## Methods

### Experimental Settings

**Figure 6:**
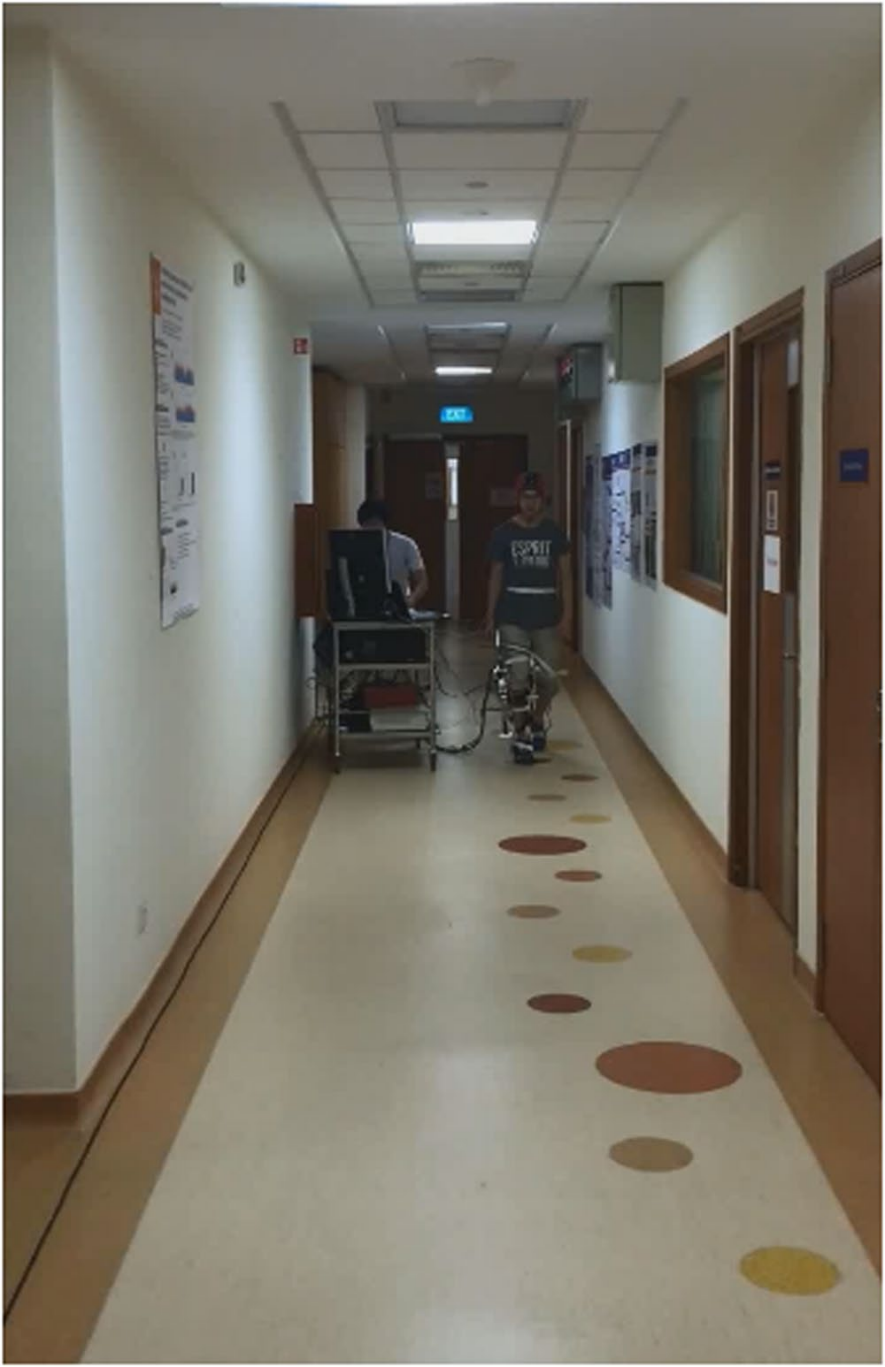
The environment where walking experiments were conducted. A triple-deck trolley accommodating data acquisition equipment and monitoring displays was pushed by an experimenter who followed alongside the participant.

Experiments were performed on a horizontal aclinal corridor approximately 70 feet long and 7 feet wide. 62-channel EEG covering entire scalp, 1-channel dipole EOG placed above and below the right eye, and 4-channel EMG placed on the surfaces of major walking-related muscles (i.e., Tibialis Anterior, Gastrocnemius Lateralis, Rectus Femoris, and Semitendinosus) of the right lower limb were used for data recording by an ANT ASA-Lab system (ANT BV, Netherlands). All channels were simultaneously recorded at a sampling rate of 1000 Hz. A compact and wearable unilateral exoskeleton was attached to the right lower limb of participants to provide assistive torque for overground walking^38^. A triple-deck trolley accommodating data acquisition equipment and monitoring displays was pushed by an experimenter who followed alongside the participant, maintaining a proper distance from the participant. A picture shot at the experimental environment is shown in Fig. 6. The informed consent from the participants in Fig. 6 was obtained for publishing the information/image in an online open-access publication.

### Protocol

Participants walked under four different conditions (1) walking with a unilateral exoskeleton, but no torque assistance is provided; (2) normal walking without unilateral exoskeleton support; (3) walking with a unilateral exoskeleton that provides low assistive torque; (4) walking with a unilateral exoskeleton that provides high assistive torque. The data of conditions (2) and (3) were used in this study. These two conditions, hereinafter, are referred to as the condition without exoskeleton and the condition with exoskeleton.

We instructed participants to walk as they normally would, without any restriction. Prior to the formal walking, participants were given practice to acquaint themselves with the walking with exoskeleton until they deemed it sufficient. Turning was manually performed by the experimenters once participants reached the end of the corridor. The recording was stopped before the turning and resumed before the participants walked forward again. Each participant achieved each walking condition three times.

### Participants

We recruited thirty healthy participants at the National University of Singapore (NUS) by advertising on the campus. All participants had normal vision or corrected-to-normal vision, and claimed that they were not suffering from or had any history of major lower limb injury or known neurological and locomotor deficits. Three of them did not complete their experiments and were excluded from the dataset. The average age of the remaining participants was 24 years old (standard deviation of 2.32 years). Their body mass index (BMI) was 22.92 ± 2.76 (mean ± standard deviation). We only recruited male participants because (1) the customized exoskeleton fits a specific range of height and weight in order to provide efficient and proper assistance; (2) female participants differ from males in strength, which might introduce bias into data analysis.

This study was reviewed and approved by the Institutional Review Board of the NUS. The procedures were carried out in accordance with the ethical standards on human experimentation. All participants gave their written informed consent forms before commencing the experiment.

### Data Processing

After downsampling to 250 Hz, adaptive filtering method was utilized to mitigate the effect of eye movement-related artifacts using EOG signal as ref.^39^. This was followed by a step of EMG artifact removal using a canonical correlation analysis-based method^40^. Subsequently, continuous EEG signal was divided into segments with two-second long. Extreme artifact-containing segments were removed by abnormal segment detection method with default parameter setting in the EEGLAB toolbox^41^. Independent component analysis (ICA) was then employed to decompose remaining EEG segments into signal sources (i.e., components). Those components relevant to artifacts were removed and the remaining components were used to reconstruct continuous artifact-free EEG signal. Finally, the artifact-free EEG signal was partitioned into gait cycles according to gait markers, which were extracted from the EMG signal^42^. Gait cycles were further visually inspected and abnormal gait cycles were removed.

### Hemispherical Asymmetry Index

Fourier transform was applied to the remaining gait cycles to obtain spectral power densities, which was followed by the logarithmic transform. After that, hemispherical effect was quantified by hemispherical asymmetry index (HAI), which was calculated by

1 $$HAI=\frac{B{P}_{L}-B{P}_{R}}{B{P}_{L}+B{P}_{R}}$$

where $B{P}_{L}$ and $B{P}_{R}$ represent band powers of the left hemisphere and right hemisphere, respectively. It is defined as leftward asymmetry when the power in the left hemisphere is greater than its counterpart in the right hemisphere. The opposite is called as rightward asymmetry.

### Source Localization

The remaining gait cycles were time-warped to the median gait length in order to align the timing of all gait cycles from all subjects. ICA was reapplied to these aligned gait cycles and performed separately for each subject, which resulted in 62 independent components (the same as the number of channels). The DIPFIT plug-in within the EEGLAB toolbox^41^ was utilized to localize best-fitting equivalent dipole locations of independent component scalp maps^43,44^. The 62-channel positions were coregistered to the surface of the boundary element model (MNI standard brain model) by warping, resizing, moving, and rotating. All components were included for the first-round clustering using k-means. The clusters with abnormal spectral characteristics and components for which the equivalent dipoles were located outside brain grey matter were removed using the plug-in of Std_selectICsByCluster (https://sccn.ucsd.edu/wiki/Std_selectICsByCluster) and the interactive interface. The remaining components were clustered again to obtain the final clusters.

### Statistical Evaluation

One-way analysis of variance (ANOVA) was used to explore whether there was a statistically significant difference between the means of hemispherical asymmetry index of cortical regions. The post-hoc paired t-test was further used to compare means when there was a significance in the ANOVA analysis. The two-tailed one-sample t-test was used to explore the power asymmetry for channel pairs. For the ERSP difference, the two-tailed two-sample t-test was utilized to evaluate whether or not spectral power was significantly different between clusters for each frequency and time point. The prerequisite assumptions of statistical methods were checked to have a valid testing result.

## Data Availability

The data analysed during the current study are available from the corresponding author on reasonable request.
